# To flee or wait and see? Response of incubating white-browed scrubwrens to information about danger

**DOI:** 10.1093/beheco/arag006

**Published:** 2026-02-02

**Authors:** You Zhou, Andrew N Radford, Robert D Magrath

**Affiliations:** Division of Ecology and Evolution, Research School of Biology, Australian National University, 46 Sullivans Creek Road, Acton, ACT 2601, Australia; School of Biological Sciences, University of Bristol, 24 Tyndall Ave, Bristol BS8 1TQ, United Kingdom; Division of Ecology and Evolution, Research School of Biology, Australian National University, 46 Sullivans Creek Road, Acton, ACT 2601, Australia

## Abstract

Animals suffer elevated predation risk during reproduction, with nesting parents having to decide whether to flee from nearby predators for their own safety or to stay and thus avoid betraying the nest's location to protect offspring. Gaining information about the source of danger is therefore crucial. From inside a nest, it might be difficult to gather relevant information visually and so acoustic signals and cues become particularly important. However, there has been little investigation of the response of nesting parents to acoustic information about different predation risks. We used a playback experiment to test how incubating female white-browed scrubwrens, *Sericornis frontalis*—which build extremely cryptic, dome-shaped nests close to the ground—respond to aerial alarm calls (warning of airborne predators), mobbing alarm calls (warning of stationary predators on or above the ground), the footstep sounds of a nest predator on the ground, and control calls of a harmless parrot. We found that incubating scrubwrens responded more to the “danger” treatments compared with the control but, contrary to expectation, rarely fled in response to any of the predatory threats. However, birds looked around more actively (more saccades) after mobbing calls compared with the other playbacks, perhaps because mobbing calls do not indicate the specific location of danger and so additional information gathering is valuable. Incubating scrubwrens can therefore recognize potential danger by sounds, but evaluated risk from within the nest rather than immediately fleeing, suggesting that they prioritize nest crypsis over other anti-predator strategies.

## Teaser

Nesting birds face a tough decision: flee from predators for their own safety or stay hidden to protect their young. This is harder when their view is blocked by nest materials. Incubating white-browed scrubwrens could use sounds to detect danger, including alarm calls and predator footsteps. They looked out more if they cannot tell the predator’s location by sounds, but overall stayed hidden to avoid nest exposure, which may enhance the chance of young survival.

## Introduction

Nesting is a critical time for animal reproduction, with high predation pressure on both nest contents and adults attending those nests (Magnhagen 1991; Deeming 2023). Eggs and dependent young within nests are vulnerable due to their immobility and, for predators, they are an easy source of protein and calories (Pike et al. 2016; Menezes and Marini 2017). Consequently, predation is the major cause of egg and offspring mortality in nests of many fish, reptiles, mammals and birds (Thompson III, 2007; Lehtonen et al. 2013; Pike et al. 2016; Seltmann et al. 2017). In addition, parents attending nests face increased predation risk, as they invest more time and energy on caring for offspring and less on vigilance against personal threats (Ibáñez-Álamo et al. 2015). Furthermore, the concentrated activity around nests and extended time parents spent incubating, brooding, roosting and feeding make them easier targets for predators who learn their routines or directly depredate the adults on the nest (Ellis-Felege et al. 2013; Ibáñez-Álamo et al. 2015; Delaney and Janzen 2020). However, compared with offspring, there has been less study of the predation risks to nesting adults during the reproductive process (Lima 2009; Ibáñez-Álamo et al. 2015).

Nesting birds use various anti-predator strategies, including shortening of vulnerable nesting periods, building the nest in places inaccessible to predators and nest defense (Lima 2009; Mainwaring et al. 2015). In areas with higher predation risk, birds may reduce clutch sizes to shorten the duration of the reproductive cycle and so minimize predation (Zanette et al. 2011; Callan et al. 2019). In addition, nesting in inaccessible locations, such as tree hollows or burrows, serves as a physical barrier against predation (Rudolph et al. 1990; Harper and Batzli 1996; Mullin and Cooper 2002). Furthermore, when there are predators around, some species actively guard their nests, deterring predators from approaching (Slack 1976; Steinhart et al. 2005; Portman 2019; Redmond et al. 2020). However, physical barriers may not prevent access by all predators, and active defense can be risky, costly or ineffective, so another general strategy is to reduce the chance that predators find nests in the first place (Montgomerie and Weatherhead 1988; Borgmann and Conway 2015).

Birds reduce the risk that predators will find nests by making them difficult to see and by reducing activities that could betray their location (Skutch 1949; Borgmann and Conway 2015). Better concealed nests, such as those that are small, camouflaged or hidden by greater foliage density, result in increased reproductive success in many species (Martin and Roper 1988; Hansell 2000; Colombelli-Négrel and Kleindorfer 2009; Liu et al. 2021). To maintain nest crypsis, some species decrease activities at the nest in areas with higher predation risk, by reducing on- and off-bout rate during incubation or feeding rate during the nestling stage (Ibáñez-Álamo and Soler 2012; Morosinotto et al. 2013; Mutzel et al. 2013; Hua et al. 2014). Lower feeding rates can be achieved by reducing clutch size or increasing the amount of food delivered during each visit (Ghalambor and Martin 2001; Strickland and Waite 2001; Raihani et al. 2010; Mariette and Griffith 2012). Moreover, because vocalisations such as mobbing calls from parents and begging calls from nestlings can attract predators (Krams et al. 2007; Haff and Magrath 2011; Bonnington et al. 2013), both parents and the young tend to stay silent at the nest when predators are nearby (Davies et al. 2004; Haff and Magrath 2013; Węgrzyn and Leniowski 2015; Maziarz et al. 2019).

If adopting the cryptic nest strategy, parent birds face a tradeoff between reducing their own vulnerability and that of their offspring when predators are close to the nest, which requires assessing current risks (Dale et al. 1996). For their own survival, parents might reduce their own risk by fleeing from the nest if there is a nest predator nearby. However, any activity around the nest–including fleeing from it–could betray the nest location, leading to higher risk for eggs or nestlings (Martin et al. 2000; Şahin Arslan and Martin 2024). Alternatively, parents could choose to stay in the nest to maintain nest crypsis, but they are then vulnerable if the predator does find the nest. Gaining information about danger is therefore important for parents to make the decision about whether to flee or stay (Schneider and Griesser 2013). Overall, parents should have fine-tuned responses according to their assessment of danger (Munoz and Blumstein 2012; McLachlan and Magrath 2020), and should flee the nest only when they are in immediate danger (Montgomerie and Weatherhead 1988; Graham and Shutler 2019). However, experimental tests of this prediction are rare.

Acoustic information about danger becomes particularly valuable when birds are visually restricted in a cryptic nest (Stevens 2013). Such information could come from alarm calls, including functionally referential alarm calls that warn of different types of predators (Marler 1957; Macedonia and Evans 1993; Caro 2005; Gill and Bierema 2013; Manser 2022). Functionally referential calls include aerial alarm calls for predators in flight (Wheeler and Fischer 2012), mobbing alarm calls for perched or terrestrial predators (Carlson and Griesser 2022) and alarm calls to specific type of predators, such as to snakes or to cuckoos (Suzuki 2015; Feeney et al. 2025). Nestlings respond appropriately to different alarm calls (Magrath et al. 2007a; Fasanella and Fernández 2009; Magrath et al. 2010), but there has been little study of the response of adult birds in the nest: one exception is that incubating Japanese tits, *Parus minor*, flee from the nest to alarm calls specifically for snakes but not to crows, because only snakes can access their nests in tree hollows (Suzuki 2015). Individuals can additionally use sounds produced by predators, such as calls or sounds of locomotion, to assess the risk of predation (Magrath et al. 2007a; Schneider and Griesser 2013; Amorim and Dias 2021), yet such direct sounds about danger have drawn surprisingly little attention. Attending to acoustic signals and cues of danger is likely to be especially important during incubation, when parents can spend long periods in the nest, and is therefore an important time for parents to balance their own survival against that of their offspring.

**Figure 1:**
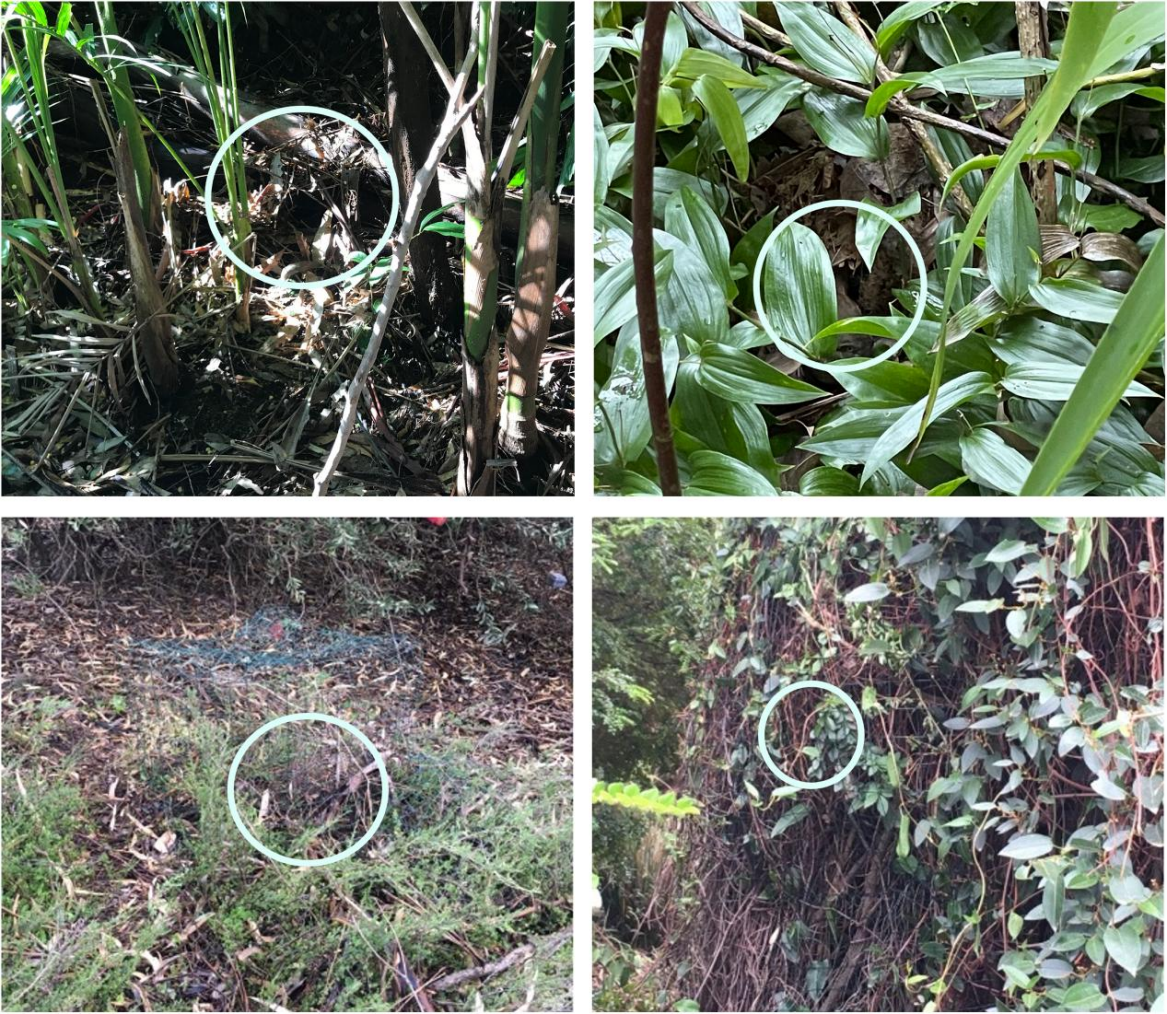
Photographs of scrubwren nests (Credit: You Zhou). The nest entrances are indicated by white circle. Each lower photograph also shows the cage installed to protect the nest against predators.

In this study, we assessed the use of acoustic information about danger by incubating white-browed scrubwrens, *Sericornis frontalis*. White-browed scrubwrens are an ideal model because females build cryptic domed nests close to the ground—often under leaf litter or low plants, with overhead cover—and rely largely on nest crypsis to avoid predators during nesting (Higgins et al. 2001; Magrath et al. 2000; Fig. 1). Nest predation increases from about 1% per day during incubation to 4% to 5% per day during the nestling stage (Platzen and Magrath 2004), probably reflecting increased cues of the nest as it progresses, such as greater activities of parents feeding nestlings and the begging of nestlings, with predators shown experimentally to be attracted by nestling calls (Haff and Magrath 2011). Incubating and brooding females are also vulnerable to predation, with 6 of 114 females followed for 1 or more breeding attempts thought to have been killed at the nest during incubation and 7 thought killed when nestlings were <6 days old (Magrath, unpublished data 1992 to 1998; individually marked females only; *n* = 679 nesting attempts with known fate). Scrubwren nestlings become quiet after playback of alarm calls or footsteps from nest predators (Platzen and Magrath 2004, 2005; Magrath et al. 2007a; Haff and Magrath 2010; Haff and Magrath 2013), suggesting that these sounds could be valuable sources of information for incubating females as well. We therefore broadcast scrubwren alarm calls and the sound of a predator's footsteps to incubating females, along with the sound of a harmless parrot (as a control). We predicted that the females would respond more to sounds of danger, rather than call in general, and would be more likely to flee the nest, or become more vigilant, to sounds indicating greater personal danger. In particular, we predicted that scrubwrens would stay in the nest, with minimal responses, after playback of aerial alarm calls, as airborne predators are unlikely to spot the nest from above. By contrast, they would be more likely to be alert for longer, or even flee, to playback of mobbing calls and the sound of a predator's footsteps, which indicate potential immediate risks from nearby terrestrial predators.

## Methods

### Study species and site

White-browed scrubwrens are small (13 to 14 g), cooperatively breeding passerines native to southeast Australia (Higgins et al. 2001). They typically breed in pairs or in groups consisting of a dominant pair and 1 or more subordinate males (Magrath and Whittingham 1997). Their nests are small (around 15 cm in diameter), cryptic, domed structures, hidden in vegetation or under leaf litter on or near the ground (Magrath et al. 2000; Fig. 1). Females usually lay a clutch of 3 eggs, which they alone incubate for an average of 18 days while males seldom go to the nest during incubation (Magrath et al. 2000). After hatching, both members of the dominant pair feed the offspring throughout the nestling and fledgling stages, and subordinate males often do so too (Magrath and Whittingham 1997; Magrath et al. 2000; Leedman and Magrath 2003).

Scrubwrens produce different alarm calls to warn of different threats (Higgins et al. 2001). When predatory birds are airborne, scrubwrens produce aerial alarm calls composed of repeated elements, each about 100 ms, with a mean ± SD peak frequency of 7.1 ± 0.4 kHz (Leavesley and Magrath 2005; Magrath et al. 2007b). A greater number of elements in aerial alarm calls indicates a greater urgency of predation risk, and calls consisting of 3 or 4 elements usually prompt immediate flight to cover by foraging scrubwrens (Leavesley and Magrath 2005; Fallow and Magrath 2010). For perched predators, or those on the ground, scrubwrens produce mobbing alarm calls which recruit conspecifics to deter the predators (Platzen and Magrath 2005). Mobbing calls also consist of repeated elements, each about 120 to 180 ms with a peak frequency of 6.5 to 8.0 kHz (Platzen and Magrath 2005), and mobbing calls can continue as long as a threat is nearby (pers. obs.).

We studied scrubwrens in the Australian National Botanic Gardens (−35.279°S, 149.109°E) in Canberra from August to December 2022. Scrubwrens breed throughout most of the 40 ha Gardens, which contain rainforest, natural woodland, native plant gardens and lawns. The population is subject to long-term studies and accustomed to people (Magrath et al. 2000; Platzen and Magrath 2004; Haff et al. 2015; Zhou et al. 2024). The primary nest predator of scrubwrens in the area is the pied currawong, *Strepera graculina*, a crow-like passerine weighing around 300 g that uses both visual and acoustic information to hunt for eggs, nestlings and even adult scrubwrens in the nests (Higgins et al. 2001; Magrath et al. 2010; Haff and Magrath 2011). Other nest predators at the study site include gray butcherbirds, *Cracticus torquatus*, Australian water dragons, *Intellagama lesueurii*, and eastern brown snakes, *Pseudonaja textilis* (Platzen and Magrath 2005; Haff and Magrath 2010).

### Experimental design and specific predictions

To test how incubating female scrubwrens assess information about danger, we broadcast 4 acoustic treatments to birds at the nest; each of the 16 focal females received all 4 treatments in a matched design. The playbacks were: (1) scrubwren aerial alarm calls; (2) scrubwren mobbing alarm calls; (3) the sound of footsteps of pied currawongs walking on leaf litter; and (4) as a control, contact calls of crimson rosellas, *Platycercus elegans*, which are harmless parrots commonly found at the study site.

We predicted that incubating scrubwrens would respond to the 4 playback treatments according to their context of production. First, aerial alarm calls warn of flying predators. As birds in their ground-level, dome-shaped nests are invisible from above, aerial alarm calls indicate an indirect threat to incubating females. Therefore, we predicted that females would stay in the nest and show minimal responses. Second, mobbing alarm calls warn of perched or terrestrial predators which might target the nest. The location of such predators is unknown, so we predicted that incubating females should look around more frequently than when responding to other treatments, and leave the nest for personal safety or to allow them to join in with mobbing. Third, currawong footsteps are sounds directly from the predator, which indicates something moving near the nest. We predicted that incubating female scrubwrens should either immediately flee the nest or check their surroundings, especially in the direction of the sound source. Last, rosella contact calls are not related to any potential danger and so incubating females should have no response to this control sound. In addition, we predicted longer response duration to the treatments that females perceived as indicating higher levels of predation risk; that is, a longer response to mobbing alarm calls and currawong footsteps than to aerial alarm calls, and the shortest to rosella calls.

### Sound file preparation

**Figure 2:**
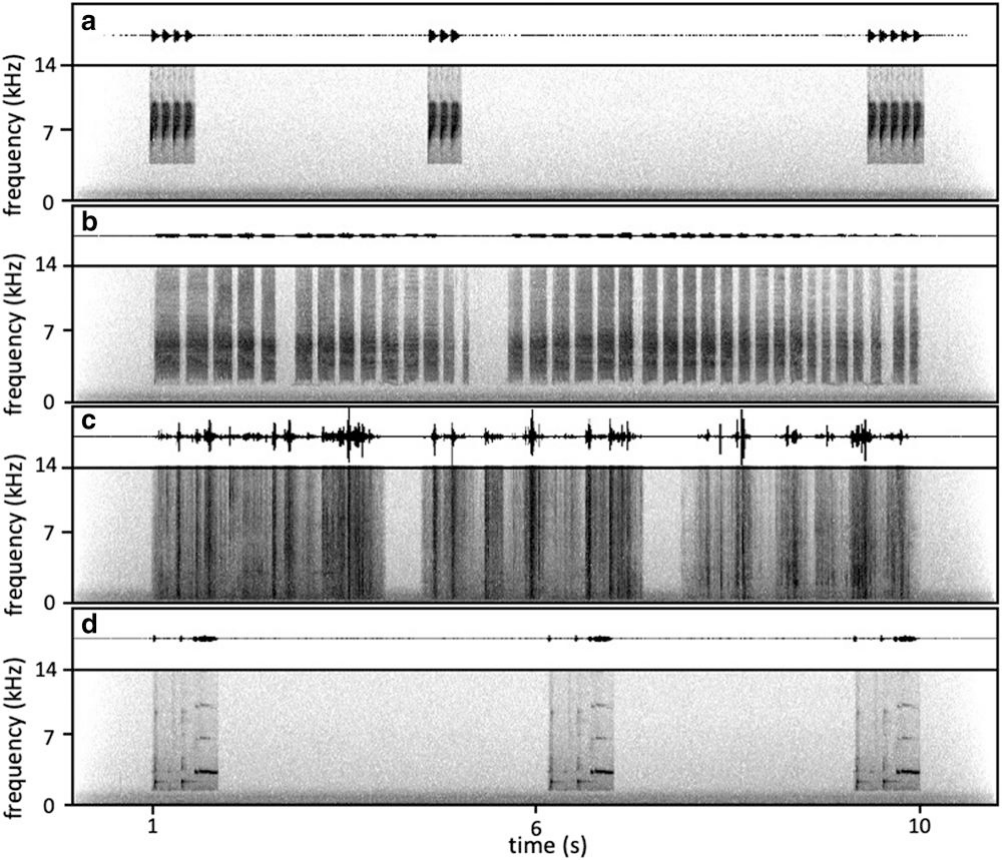
Examples of playback sounds: a) scrubwren aerial alarm calls; b) scrubwren mobbing alarm calls; c) pied currawong footsteps; and d) crimson rosella contact calls. The waveform is shown above and the spectrogram below in each case. The amplitude is on a linear scale and expressed as the uncalibrated digital amplitude with a range from −32,768 to 32,768, which is the digital range of 16-bit wave tracks. Spectrograms were prepared in Raven Pro 1.6 (Hann window type, 512 window sample size and 50% overlap).

We prepared 16 unique sets of playback tracks, 1 for each of the 16 nests, to avoid pseudoreplication. Each playback lasted 10s, to ensure that differences in duration could not explain any differences in response among treatments. In nature, individual aerial alarm calls are usually short, with few elements and each call less than 1s (Leavesley and Magrath 2005), but they can be repeated each time a predator flies (personal observations). Mobbing calls are variable in duration, sometimes continuing with a predator's presence and lasting more than 30s (personal observations). Footstep sounds are likely to be variable in duration, depending on movement and substrate. Individual rosella “bell” contact calls typically last less than 1s, but are often repeated (personal observations). For realism, we therefore included 3 repeats of aerial alarm calls with 3 to 5 elements and rosella contact calls in 10s, whereas mobbing calls and footsteps continued for the full 10-s playback (Fig. 2). In this way, we present each call type within a natural range of call rate, so the experiment is ecologically relevant, while also keeping all the treatment playbacks with matched duration. To avoid any effect of an abrupt start to playbacks, a unique 12-s ambient sound was mixed into each set of treatments, with the first second of ambient sound fading in and the last second fading out. The 16 unique ambient sounds were recorded under relatively quiet conditions in the Gardens, with no distinct foreground sounds such as bird calls or nearby human noise. The ambient sounds were always at least 15 dBA lower in sound pressure level (SPL) than the loudest components of the treatments. All playback tracks were prepared using Adobe Audition 2022.

We recorded both aerial and mobbing alarm calls from male scrubwrens in the same group as the focal female. The calls were recorded using a Tascam DR-100MKIII recorder and a hand-held Sennheiser ME66 directional microphone (sampling at 44.1 kHz and 16 bits). We prompted aerial alarm calls with a gliding sparrowhawk model (Magrath et al. 2015). From each recording, we chose 1 element of an aerial alarm call of high quality and repeated it to make multielement aerial alarms that indicated a nearby flying predator. The interval between elements within a call was consistent within each playback and within the range of natural aerial alarm calls (35 to 40 ms; Leavesley and Magrath 2005). Each playback included 3 aerial alarm calls, with 8 playbacks consisting of 4-, 3- and 5-element aerial alarm calls in sequence (Fig. 2a) and the other 8 consisting of 4-, 5- and 3-element calls in sequence. The intervals between the 3 repeats were randomly selected for each playback track. We prompted mobbing calls using a snake model combined with playbacks of mobbing calls from individuals unfamiliar to the focal group (Platzen and Magrath 2005). We first found the focal group in the territory and set up the snake model with the recording system nearby. Once the targeted male was attracted to the snake model, we recorded the mobbing calls and chose a cut of 10s where there was just a single male calling (Fig. 2b). The sound of currawong footsteps walking on natural leaf litter were the same clips previously used in a study of nestlings (Magrath et al. 2007a). In brief, a Sennheiser ME66 directional microphone was put near the ground and connected with a 15 m cable to a Marantz PMD670 digital recorder (sampling at 44.1 kHz and 16 bits). Currawongs were attracted within 5 m of the microphone using food placed on the ground in an area of leaf litter. We cut out clips of clear footsteps for 3s and put together 3 different footstep clips with about 0.3s interval between each to make a 10-s playback track (Fig. 2c). Rosella contact calls were recorded under natural conditions by following individuals and recording them with a Marantz PMD670 digital recorder and a Sennheiser ME66 directional microphone (sampling 44.1 kHz and 16 bits; Magrath et al. 2007a). We made each rosella playback track by including 3 repeats of the same call within the 10s (Fig. 2d).

We broadcast all the playback treatments at 55 dBA SPL at 1 m, as used in playbacks to nestlings during previous studies (Platzen and Magrath 2005; Magrath et al. 2007a). The playback equipment consisting of a Rokono mini-loudspeaker connected to an Edirol R-05 HR digital recorder using a 12 m cable. We kept amplitudes constant to investigate the incubating bird's interpretation of different sounds, independently of their amplitude. We calibrated the playback sounds by re-recording the playbacks at 1 m along with a calibration tone that had its amplitude measured with the Brüel & Kjær 2240 sound level meter. The playbacks of treatments and tone were then measured in Raven Pro 1.5 using the Average Power function, and playback tracks were adjusted using the Amplify function to achieve the target broadcast amplitude. We measured specifically the Average Power of the loudest call elements and the overall average of footsteps since they did not have discrete elements.

### Field playback methods

We carried out playbacks from October to December 2022. Nests were found by observing females carrying nesting material or returning to the nest during incubation. All nests were protected using large green garden mesh wire “cages”, which allowed free access by scrubwrens but not large predatory birds (Haff and Magrath 2010, 2011). The mesh material was very thin-gauge wire, placed at least 30 cm away from the nest, which was typically integrated with the surrounding vegetation and was difficult to see at a distance (Fig. 1). Birds appear to treat these cages as part of the surrounding vegetation, often perching on wire when arriving or leaving the nest (Haff and Magrath 2010; personal observations). It seems unlikely that incubating birds perceive that cages offer safety. To record the response of focal birds at the 10 nests that were partly or fully covered by vegetation, we placed an endoscope around 20 cm outside the entrance. If ambient light was insufficient and extra light was required for video recording (14 playbacks at 5 nests), the white light from the endoscope was turned on as soon as the female returned to the nest to allow habituation. At the remaining 6 nests where the entrances were fully visible from 1 m, we placed a Panasonic HC-V770M video camera at least 1 m from the entrance. For playbacks, we put the loudspeaker 1 m from the nest at an angle of more than 90° from the nest entrance so that the focal female could not see the sound source when sitting within the nest. To confirm that the playback files were broadcast smoothly, we also recorded acoustic environment at the nests using a camouflaged microphone put about 20 cm outside the nest entrance, connected to a programmable outdoor audio recorder (Bioacoustic Recorder [BAR] from Frontier Labs, 2017, sampling at 44.1 kHz and 16 bits). To minimize disturbance and allow habituation, we set up the endoscope and microphone at least 1 day before the first playback and removed them only after the entire set of playbacks was completed, during times when the incubating females were off the nest foraging. The observer remained at least 7 m from the nest after installing equipment, at which distance the birds appeared to ignore our presence. Since the playback equipment and Panasonic cameras were not waterproof, they were set up before each playback when the female was off the nest, and removed 10 min after the playback. The cameras and loudspeakers appeared to be ignored by the birds.

We broadcast playbacks at least 10 min after the female returned to the nest, and only if there had been no alarm calls or other sounds of danger for at least 5 min before the playback, so the female was sitting inside the nest and not being vigilant. After the playback, we recorded the response of the focal bird for at least 6 min. To minimize the impact of playbacks on scrubwrens, at most 2 trials were conducted on each nest per day, with a minimum 2-h interval. Females received the 4 treatments over 2.8 ± 1.3 days (mean ± SD; range 2 to 7 days). We used a complete block design so that each of the 4 treatments occurred 4 times in each order.

### Scoring response to playback

We scored videos frame-by-frame to measure the birds' behavior after playback. The videos were scored blindly to treatment type and nest identity; the time of playback initiation was first noted, and then the sound was muted and the names of videos were re-assigned by others before scoring. We used 3 measures of response: (A) categorical response; (B) duration of response; and (C) number of saccades (discrete head movements). (A) The categorical response was scored as the greatest ranked response shown after playback, where: 0 = no response, the bird remained in the same state, relaxed and incubating the eggs, as before the playbacks; 1 = look, the bird stayed in the nest and moved her head around; 2 = look out, the bird moved her head out of the nest entrance and looked around; 3 = flee, the bird left the nest. (B) The duration of response was measured as the time from the onset of response until the focal female resumed her original, relaxed state as before the playback, and is likely to be an indication of the degree of risk the incubating female perceived from the playbacks (Beauchamp 2015). (C) The number of saccades was the number of discrete head movements for females that stayed in the nest, and is an indication of how frequently the female's gaze direction changed (Beauchamp 2015; Tyrrell et al. 2015; Land 2019). For both response duration and number of saccades, we excluded cases where the birds showed no response (*n* = 10 trials) and those where they fled (*n* = 7 trials), because none returned to the nest within the 6-min sample period. Although playbacks were conducted when males were not around, males approached and mobbed after playback in 4 cases: in 2 cases after the female had already left the nest (one to mobbing call playback and one to footstep playback), and in 2 cases while the female remained in the nest but had already put her head out (both to mobbing playbacks). Since male presence occurred only after the female had exhibited her greatest categorical response, it would not affect measurement of categorical responses. For response duration and number of saccades, excluding the 2 cases where males arrived while the female remained in the nest led to similar results to the complete dataset (Supplementary Results).

### Statistical analysis

We conducted all statistical analyses in R v 4.2.0 (R Core Team 2022). We used cumulative link mixed models (CLMMs) to analyse the categorical responses of incubating scrubwrens to each playback treatment. CLMMs are appropriate for analysing ordinal responses, which arranges the response strength without implying quantitatively equal intervals between categories (Agresti 2007). The categorical response was ranked into 4 ordered levels: 0 = no response, 1 = look, 2 = look out, 3 = flee. We fitted 2 CLMMs to test differences in categorical response. The first model included, as the fixed factor, all playback treatments (scrubwren aerial alarm call, scrubwren mobbing call, currawong footsteps, rosella contact call). The second model excluded the control (rosella call) and tested for the differences among the 3 danger treatments only, because the “no response” category occurred only in control playbacks, making the distribution of responses to the control very different to those to the danger treatments. In both models, we also included whether a camera light was turned on or off as a fixed factor to control for a possible light effect, and random factors were the focal individual and treatment order. We conducted the CLMMs using the “clmm()” function of the “ordinal” package (Christensen 2023), with a probit link function and an equidistant threshold. We used the “Anova()” function of the “car” package (Fox and Weisberg 2019) to test the significance of each fixed factor. If the treatment was found to be significant overall, then we used the “emmeans()” function of the “emmeans” package (Lenth and Piaskowski 2018) to conduct pairwise comparisons between treatments.

We used linear mixed models (LMMs) to analyse the response duration and the number of saccades during the responses (both quantitative variables). The fixed factors were treatment (scrubwren aerial alarm call, scrubwren mobbing call, currawong footsteps, rosella contact call) and camera light status (on or off). The random factors were the focal individual and treatment order. In addition, we included response duration in the model examining the number of saccades to control for the possibility that the number of saccades was due simply to the response duration. Both quantitative measurements were logarithmically transformed to improve fit for the models. We conducted LMMs using the “lmer()” functions of the “lme4” package (Bates et al. 2015), while using the “Anova()” function of the “car” package (Fox and Weisberg 2019) to test the significance of each fixed factor. We also used the “emmeans()” function of the “emmeans” package (Lenth and Piaskowski 2018) to compare between treatments if that fixed factor was found to be significant overall.

### Ethical note

The study was approved by the Australian National University Ethics Committee (protocol A2022/15) and designed to minimize any adverse effects on the birds. All the nests were caged using mesh wire, which allowed scrubwrens free access but prevented large predators from accessing the nests. To mitigate the potential impact of alarm-call and predator-footstep playbacks, each scrubwren received at most 2 trials per day, with a minimum of 2 h between them to allow the female to finish at least 1 incubation bout (average on-bout duration is about 50 min and off-bout duration about 25 min). Birds resumed relaxed incubation behavior (no looking) within 6 min after the playbacks, except for 5 cases where birds fled. In all cases that birds fled, they went back to the nest and continued incubating after around 20 min, which is a normal period to be off the nest.

## Results

**Figure 3:**
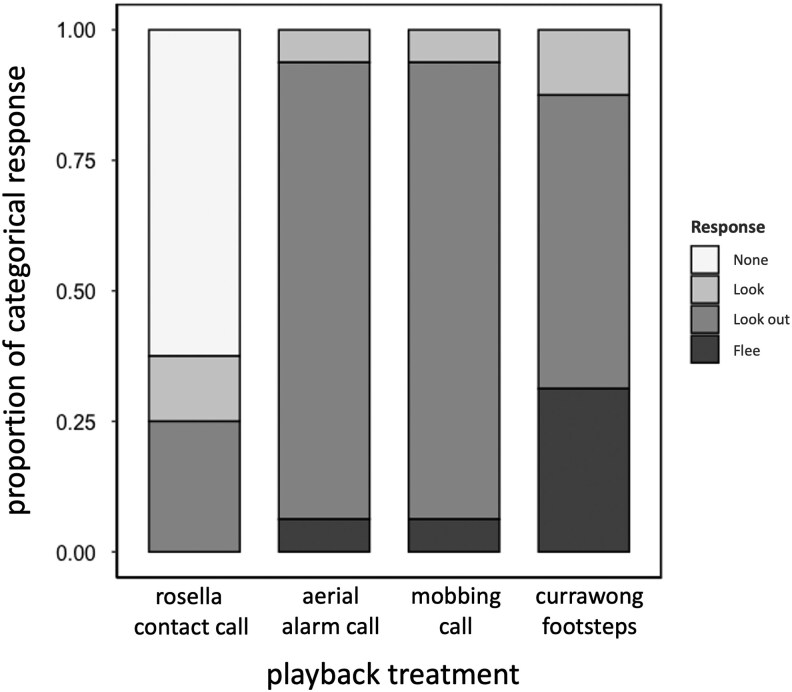
The proportion of incubating female scrubwrens that responded in different ways to the 4 playback treatments. The results of statistical analyses are presented in Table 1; *n* = 64 playbacks to 16 focal birds.

Incubating females usually looked out of the nest after danger treatments but not the control (categorical response, controlling for camera light status: CLMM: χ^2^ = 69.990, df = 3, *P* < 0.001; Table 1a; Fig. 3). Most incubating scrubwrens showed no response to control rosella calls, but had a similar looking response to all 3 sounds of danger. Considering just the 3 danger treatments, there was a nonsignificant trend (χ^2^ = 4.470, df = 2, *P* = 0.107) for more fleeing in response to the currawong footstep sounds (5/16 trials) than either the aerial or mobbing alarms (1/16 trials in each case; Table 1b; Fig. 3).

**Table 1:** Output from CLMMs investigating categorical responses of incubating female scrubwrens to a) all 4 playback treatments and b) the 3 danger treatments; results shown in Fig. 3.

Fixed effects	Estimate ± SE	χ^2^	df	*z* ratio	*P*
**(a) CLMM (all 4 treatments)**					
Camera light (on—off)	−0.167 ± 0.060	…	1	−2.806	**0.005**
Treatment	…	69.990	3	…	**<0.001**
Treatment (rosella—aerial)	−0.441 ± 0.063	…	1	−6.957	**<0.001**
Treatment (rosella—mobbing)	−0.442 ± 0.064	…	1	−6.957	**<0.001**
Treatment (rosella—footsteps)	−0.515 ± 0.063	…	1	−8.245	**<0.001**
Treatment (aerial—mobbing)	−0.001 ± 0.068	…	1	−0.016	1.000
Treatment (aerial—footsteps)	−0.074 ± 0.067	…	1	−1.111	0.683
Treatment (mobbing—footsteps)	−0.073 ± 0.067	…	1	−1.091	0.695
**(b) CLMM (danger treatments only)**					
Camera light (on—off)	−0.422 ± 0.522	…	1	0.809	0.409
Treatment	…	4.470	2	…	0.107
Treatment (aerial—mobbing)	0.007 ± 0.079	…	1	0.082	0.996
Treatment (aerial—footsteps)	0.263 ± 0.122	…	1	2.154	0.079
Treatment (mobbing—footsteps)	0.256 ± 0.125	…	1	2.057	0.099

Significant effects are shown in bold. *n* = 64 playbacks to 16 focal birds.

**Figure 4:**
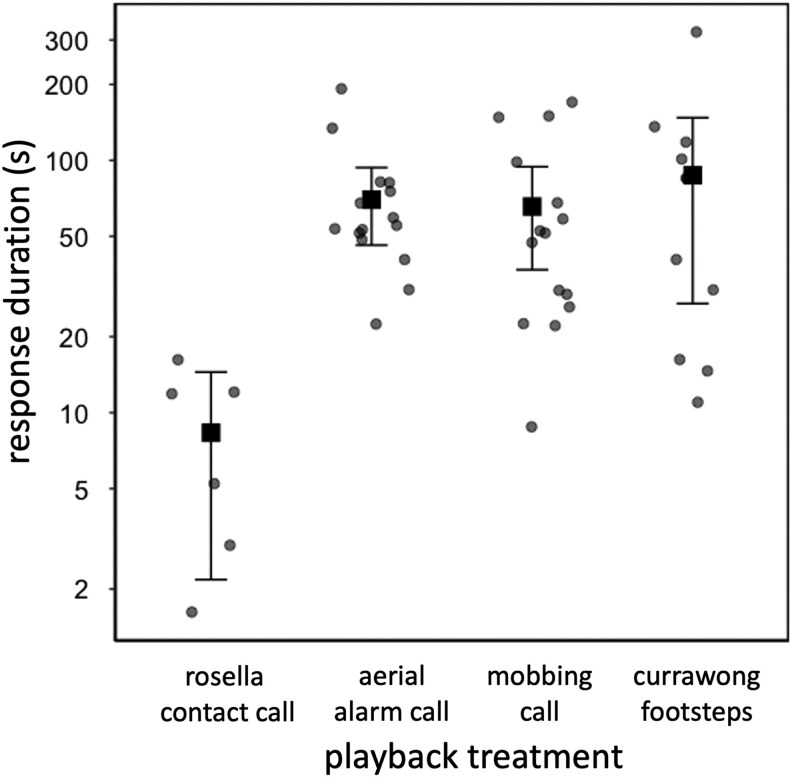
Duration of looking response by incubating female scrubwrens to different playback treatments. Each round point represents a different focal female; square points and error bars indicate means and 95% CI. Note that the *y* axis is on a log scale. The results of statistical analysis are shown in Table 2; *n* = 6 for rosella contact calls, *n* = 15 for scrubwren aerial alarm calls, *n* = 15 for scrubwren mobbing calls, and *n* = 11 for currawong footsteps; cases when the focal female either did not respond or fled to the playback are excluded.

If females stayed in the nest after playbacks, they responded for longer to the danger treatments than the control (variation among treatments: LMM: χ^2^ = 44.118, df = 3, *P* < 0.001; Table 2; Fig. 4). However, contrary to our prediction, there was no significant difference in the duration of response among danger treatments (Table 2).

**Table 2:** Output from LMM investigating the duration of looking response by incubating female scrubwrens to the playback treatments, shown in Fig. 4.

Fixed effects	Estimate ± SE	χ^2^	df	*t* ratio	*P*
Intercept	1.821 ± 0.326	…	…	…	
Camera light (on—off)	0.598 ± 0.308	…	1	−1.945	0.066
Treatment	…	44.118	3	…	**<0.001**
Treatment (rosella—aerial)	−2.402 ± 0.393	…	1	−6.111	**<0.001**
Treatment (rosella—mobbing)	−2.183 ± 0.395	…	1	−5.523	**<0.001**
Treatment (rosella—footsteps)	−2.384 ± 0.427	…	1	−5.588	**<0.001**
Treatment (aerial—mobbing)	0.219 ± 0.287	…	1	0.762	0.870
Treatment (aerial—footsteps)	0.017 ± 0.320	…	1	0.054	1.000
Treatment (mobbing—footsteps)	−0.201 ± 0.319	…	1	−0.631	0.921

Duration was logarithmically transformed. Significant effects are shown in bold. *n* = 6 for rosella contact calls, *n* = 15 for scrubwren aerial alarm calls, *n* = 15 for scrubwren mobbing calls, and *n* = 11 for currawong footsteps; cases when the focal female either did not respond or fled to the playback are excluded.

**Figure 5:**
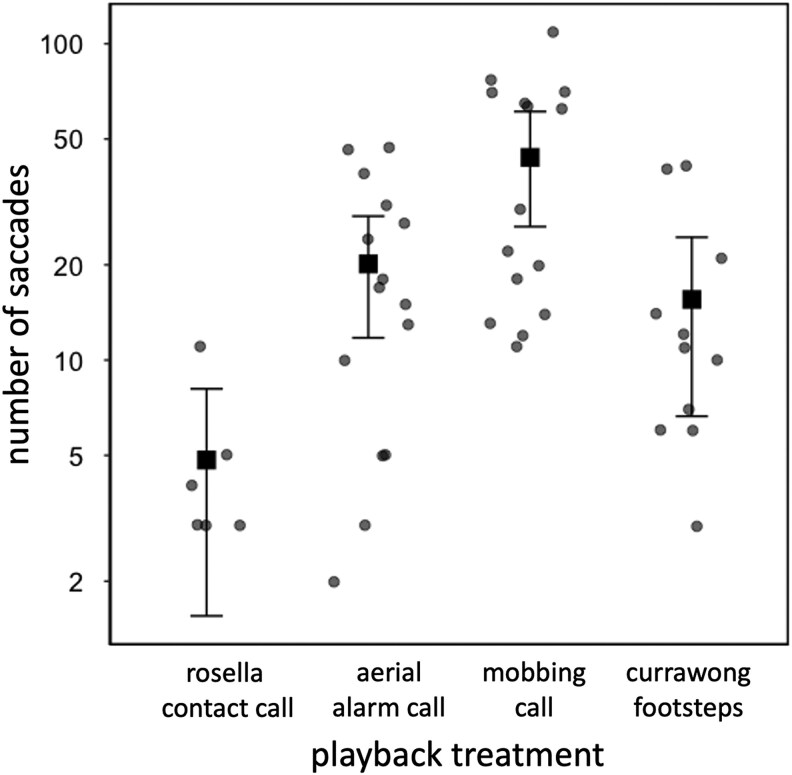
Number of saccades by incubating female scrubwrens responding to different playback treatments. Each round point represents a different focal female; square points and error bars indicate means and 95% CI. Note that the *y* axis is on a log scale. The results of statistical analysis are shown in Table 3; *n* = 6 for rosella contact calls, *n* = 15 for scrubwren aerial alarm calls, *n* = 15 for scrubwren mobbing calls, and *n* = 11 for currawong footsteps; cases when the focal female either did not respond or fled to the playback are excluded.

When being vigilant, the number of saccades (discrete head movements) made by incubating female scrubwrens was affected by treatment (LMM: χ^2^ = 23.514, df = 3, *P* < 0.001; Table 3; Fig. 5). There was a significantly greater number of saccades in response to mobbing calls compared with aerial alarm calls (*t* = 3.517, *P* = 0.008), currawong footsteps (*t* = 3.423, *P* = 0.010) and rosella controls (*t* = 0.320, *P* = 0.014). There was no significant difference between aerial alarm calls and currawong footsteps, as predicted, nor between aerial alarm calls or currawong footsteps and the rosella control, perhaps because only a small subset of females responded at all to the control (Table 3).

**Table 3:** Output from LMM investigating the number of saccades by incubating female scrubwrens responding to the playback treatments, shown in Fig. 5.

Fixed effects	Estimate ± SE	χ^2^	df	*t* ratio	*P*
Intercept	0.793 ± 0.420	…	…	…	
Camera light (on—off)	0.078 ± 0.378	…	1	0.208	0.838
Response duration	0.279 ± 0.149	…	1	1.871	0.069
Treatment	…	23.514	3	…	**<0.001**
Treatment (rosella—aerial)	−0.722 ± 0.527	…	1	−1.368	0.528
Treatment (rosella—mobbing)	−1.637 ± 0.507	…	1	−3.230	**0.014**
Treatment (rosella—footsteps)	−0.648 ± 0.545	…	1	−1.190	0.638
Treatment (aerial—mobbing)	−0.916 ± 0.260	…	1	−3.517	**0.008**
Treatment (aerial—footsteps)	0.073 ± 0.291	…	1	0.251	0.994
Treatment (mobbing—footsteps)	0.989 ± 0.289	…	1	3.423	**0.010**

Number of saccades is logarithmically transformed. Significant effects are shown in bold. *n* = 6 for rosella contact calls, *n* = 15 for scrubwren aerial alarm calls, *n* = 15 for scrubwren mobbing calls, and *n* = 11 for currawong footsteps; cases when the focal female either did not respond or fled to the playback are excluded.

## Discussion

Incubating scrubwrens became alert to sounds that indicated predation risks, and looked around more frequently in response to mobbing calls compared with aerial alarm calls and predator footsteps. Overall, females showed similar elevated responses to scrubwren aerial alarm calls, scrubwren mobbing alarm calls and the sound of currawong footsteps for both categorial responses and response duration. Although there was a trend for birds to flee more to currawong footsteps than to alarm calls, overall the incubating birds rarely fled the nest, indicating that they prioritize gathering information and nest crypsis. Incubating scrubwrens looked around more (had more head saccades) in response to mobbing alarm calls than aerial alarm calls and predator footsteps, suggesting that they attend to call context: mobbing calls give no information on the direction of threat, as they can indicate either terrestrial or perched predators. To our knowledge, this study is the first to compare experimentally how incubating adults assess different sounds of danger, including both alarm calls from conspecifics and sounds from predators.

Incubating scrubwrens stayed in the nest but increased vigilance in response to danger cues, suggesting that they prioritize crypsis over fleeing. The results are consistent with the nest crypsis hypothesis that birds should remain in the nest until risks of predation outweigh the cost of fleeing (Montgomerie and Weatherhead 1988). Scrubwren nests are generally very well-hidden, and the low fleeing rate is consistent with females perceiving the playbacks as indicating intermediate level of risks, requiring further visual assessment. The responses of incubating scrubwrens to sounds of danger are also consistent with previous studies on anti-predator strategy of scrubwren parents and nestlings (Haff et al. 2015). In the field, incubating females rarely flush from the nest until it is almost touched (personal observations). Also, scrubwren nestlings suppressed calling but never fled the nest in response to alarm calls and currawong footsteps, even later in the nestling period when capable of early fledging (Platzen and Magrath 2005; Haff and Magrath 2010). Similarly, incubating brown thornbills, *Acanthiza pusilla*, stayed in their dome-shaped nests that were mostly in dense vegetation, and looked out from the nest in response to currawong call playbacks (Schneider and Griesser 2013). Our results also align with studies of other species that found birds tend to stay in the nest and tolerate closer predator approach in better concealed nests, such as by incubating tree swallows, *Tachycineta bicolor*, mallards, *Anas platyrhynchos*, and Canada geese, *Branta canadensis* (Albrecht and Klvaňa 2004; Miller et al. 2013; Graham and Shutler 2019). By contrast, incubating female Japanese tits, as well as the nestlings, fled the nest to snake-specific alarm calls but huddled down after general alarm calls warning of other predators. Such different responses to alarm calls might be due to the tits' use of cavity nests, which can be accessed by snakes but not other predators (Suzuki 2011, 2015).

Although the broad responses to all sounds of danger were similar, incubating females showed fine-tuned assessment by increasing head movements after playback of mobbing calls. This is consistent with our prediction that birds in the nest could gain acoustic information about predator type or location. Looking around in response to mobbing calls is common in birds (Suzuki 2012; Carlson and Griesser 2022). For example, foraging Australian magpies, *Cracticus tibicen*, actively scanned after playback of mobbing calls; they moved away from experimentally added visual barriers to scan instead of scanning in place, which was their response when the barrier was lying down (Ratnayake et al. 2021). Furthermore, mobbing calls typically indicate a nearby threat but lack precise directional information about the location of the threat, which might prompt birds to increase vigilance to locate the danger. In our study, the significantly higher number of saccades in response to mobbing calls suggests that birds in nests needed more visual information when the acoustic signal was more ambiguous about the location of threat. This contrasts with their response to aerial alarm calls and rosella contact calls, which are less relevant to scrubwrens in dome-shaped nests, and to predator footsteps, which also indicate nearby risk but provide clear directional cues.

Incubating scrubwrens looked out of the nest after playbacks of currawong footsteps, showing that they pay attention to sounds from predators themselves, not just to conspecific signals of danger. There was a trend for scrubwrens to be more likely to flee from the nest after playback of footsteps, compared with alarm calls (5/16 fled to footsteps, compared with 1/16 for each alarm treatment), but most females stayed in the nest, suggesting that most assess risk rather than assuming immediate danger. One possibility is that birds cannot recognize predators solely by their footsteps and so require additional information. We can find only one study on other bird species testing if they use the sounds of predator locomotion as a cue of danger: gregarious sparrows (Passerellidae) became alert but rarely fled to dog footsteps during foraging (Shearer and Beilke 2023). In addition, vibrations caused by predator footsteps are used as warning signs, including in arthropods such as in common angle moths, *Semiothisa aemulataria*, and termites, *Coptotermes acinaciformis,* and some vertebrates, such as red-eyed treefrogs, *Agalychnis callidryas* (Castellanos and Barbosa 2006; Oberst et al. 2017; Virant-Doberlet et al. 2019; Jung et al. 2022). Future work should therefore address whether birds more generally use information encoded in the danger cues caused by predator movement, potentially including walking and flight.

Contrary to our prediction, incubating scrubwrens also looked out with similar response duration to the aerial alarm calls as to other sounds of danger. We had assumed aerial alarm calls were not immediately relevant to birds within their cryptic nests. One possible reason is that an airborne predator might later land near the nest, posing a delayed threat to the nest and the bird inside, and so it is still worth assessing risk. Furthermore, nesting scrubwrens might increase their level of response to aerial alarm calls to compensate for the lack to visual information, even if the calls refer to a relatively low risk. Such increased responses to aerial alarm calls occur in New Holland honeyeaters, *Phylidonyris novaehollandiae*, when nectar-foraging with restricted views, compared with perching with clear views (McLachlan et al. 2019).

Our study presented each type of acoustic signal or cue in isolation, but real-world scenarios may often require nesting animals to integrate multiple and/or multisensory indicators of danger when assessing predation risks, while predators may similarly use multiple sources of information to find nests (Ibáñez-Álamo et al. 2015). For acoustic information, longer signals or a combination of several sounds about danger would provide more precise information about identity and locomotion of predators, which might prompt birds to flee nests. For example, the sound of footsteps alone might be from a nonthreatening animal, whereas the combination of mobbing calls and footstep sounds could indicate a predator, implying a higher risk of danger and prompting flight from the nest. Other sensory modalities, such as vibrations, odors and chemical cues, are also potentially valuable sources of information about predation risk, especially when animals are visually restricted in the nest (Stevens 2013; Williams et al. 2023). For instance, wood frog, *Lithobates sylvaticus*, embryos delayed hatching in the presence of chemical alarm cues that indicated predation (Rivera-Hernández et al. 2025). In the case of scrubwrens, the sound of footsteps combined with vibration of the nest might indicate an urgent condition where the predator is attacking the nest, and so might prompt incubating females to flee immediately without further looking. To develop a more comprehensive understanding of predator detection and response for nesting animals, we suggest future studies adopt a multisensory approach and consider how combinations of multiple cues about danger are assessed (Partan and Marler 2005). Furthermore, it would be valuable to assess what cues predators use in finding nests, as these again may involve sequences of acoustic cues or multisensory information. For example, predators may use visual cues at a distance, and then sounds, scents or other chemical cues to pinpoint nest location.

In summary, incubating female scrubwrens recognized potential danger from acoustic information alone, but usually responded by staying in the nest and assessing the environment, rather than fleeing. To deepen our knowledge of how prey animals adapt their reproductive strategies in response to predation pressure, we suggest investigating how the broader acoustic environment—including sounds from conspecifics, heterospecifics and predators—influences survival of nesting animals across taxa. Moreover, the challenges of visual restriction are not limited to nesting but also apply to year-round daily activities, such as foraging in dense vegetation. Future studies could examine how animals use different types of information to assess predation risk, which will help to understand the dynamic anti-predator strategies of animals under restricted environments.

## Supplementary Material

arag006_Supplementary_Data

## Data Availability

Analyses reported in this article can be reproduced using the data provided by Zhou et al., (2026).
