# Supplementary material for: To flee or wait and see? Response of incubating white-browed scrubwrens to information about danger
Source: Behav Ecol. 2026 Feb 2;37(2):arag006. doi: 10.1093/beheco/arag006 (PMC13017106; doi:10.1093/beheco/arag006)
Supplement: arag006_Supplementary_Data [file arag006_supplementary_data.docx]

**To Flee or Wait and See? Response of Incubating White-browed Scrubwrens to Information about Danger**

You Zhou, Andrew N. Radford & Robert D. Magrath

**Supplementary Material**

**Methods**

Playback trials were conducted from 5 to 18 days after the onset of incubation. We determined the laying date by either monitoring the nest since building or back-calculating from hatching date if the nest was found during incubation (using mean incubation period of 18 days; Magrath et al., 2000). For each nest, all four playback treatments were completed within 2–7 days (mean ± SD = 2.8 ± 1.3 days, with only one extreme case taking 7 days). The number of days into incubation was initially included in the full model but was later dropped, as it had no significant effect (Supplementary Table 1). Other dropped factors include distance from the observer to the nest, time from the focal female returning to the nest to the onset of the playback, and camera type (endoscope or video camera). Each of the full and reduced models were compared using the ‘anova()’ function in R. All the models showed no significant differences with or without the dropped variables.

**Supplementary Table 1.** Comparison of full models (all dropped variables included) and reduced models (all dropped variables excluded), using the ‘anova()’ function in R. The dropped variables are incubation days, distance from observer to the nest, time from the focal female returning to the nest to the onset of the playback, and camera type. In addition, response duration was included as a dropped variable in models for number of saccades.

| Model (full vs reduced model) | χ^2^ | df | P |
| --- | --- | --- | --- |
| CLMM for categorical response (treatments and control) | 18.780 | 4 | 1 |
| CLMM for categorical response (treatments only) | 1.962 | 4 | 0.742 |
| LMM for response duration | 0.275 | 4 | 0.991 |
| LMM for number of saccades | 5.604 | 4 | 0.231 |

**Results**

In 2 out of 15 mobbing call playbacks where the focal female remained in the nests, the male approached the loudspeaker and produced mobbing calls. Excluding the two cases generated qualitatively similar results in terms of response duration (Supplementary Figure 1; Supplementary Table 2) and number of saccades (Supplementary Figure 2; Supplementary Table 3).


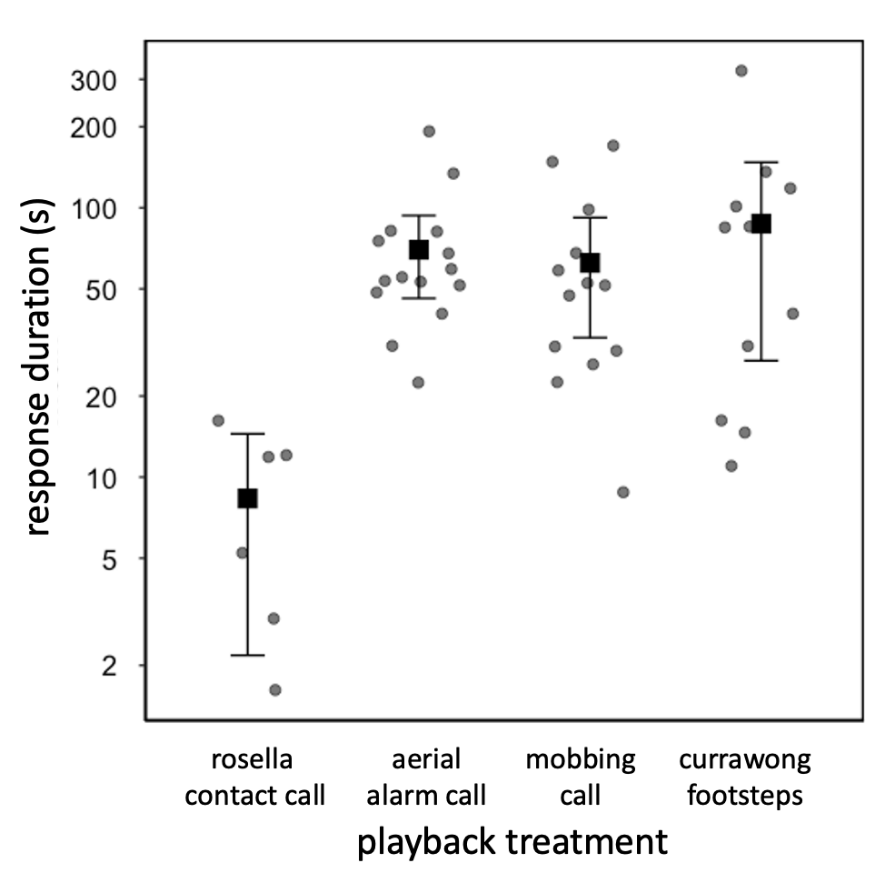


**Supplementary Figure 1.** Duration of looking response by incubating female scrubwrens to different playback treatments, excluding 2 cases where males responded to the mobbing call playbacks. Each round point represents a different focal female; square points and error bars indicate means and 95% CI. Note that the y axis is on a log scale. The results of statistical analysis are shown in Supplementary Table 2; n = 6 for rosella contact calls, n = 15 for scrubwren aerial alarm calls, n = 13 for scrubwren mobbing calls, and n = 11 for currawong footsteps; cases when the focal female either did not respond or fled to the playback are excluded.

**Supplementary Table 2.** Output from LMM investigating the duration of looking response by incubating female scrubwrens to the playback treatments, excluding 2 cases where males responded to the mobbing call playbacks. Duration was measured from the start of response until the focal female resumed its original state; duration was logarithmically transformed. Whether a camera light was turned on was also included as a fixed factor. Significant effects are shown in bold. n = 6 for rosella contact calls, n = 13 for scrubwren aerial alarm calls, n = 15 for scrubwren mobbing calls, and n = 11 for currawong footsteps; cases when the focal female either did not respond or fled to the playback are excluded.

| Fixed effects | Estimate ± SE | χ^2^ | df | t ratio | P |
| --- | --- | --- | --- | --- | --- |
| Intercept | 1.830 ± 0.327 |  |  |  |  |
| Camera light (on – off) | 0.522 ± 0.311 |  | 1 | 1.681 | 0.101 |
| Treatment |  | 41.284 | 3 |  | **<0.001** |
| Treatment (rosella – aerial) | -2.382 ± 0.402 |  | 1 | -5.925 | **<0.001** |
| Treatment (rosella – mobbing) | -2.106 ± 0.411 |  | 1 | -5.120 | **<0.001** |
| Treatment (rosella – footsteps) | -2.356 ± 0.436 |  | 1 | -5.402 | **<0.001** |
| Treatment (aerial – mobbing) | 0.276 ± 0.307 |  | 1 | 0.899 | 0.806 |
| Treatment (aerial – footsteps) | 0.026 ± 0.328 |  | 1 | 0.080 | 1.000 |
| Treatment (mobbing – footsteps) | -0.249 ± 0.340 |  | 1 | -0.733 | 0.883 |

**
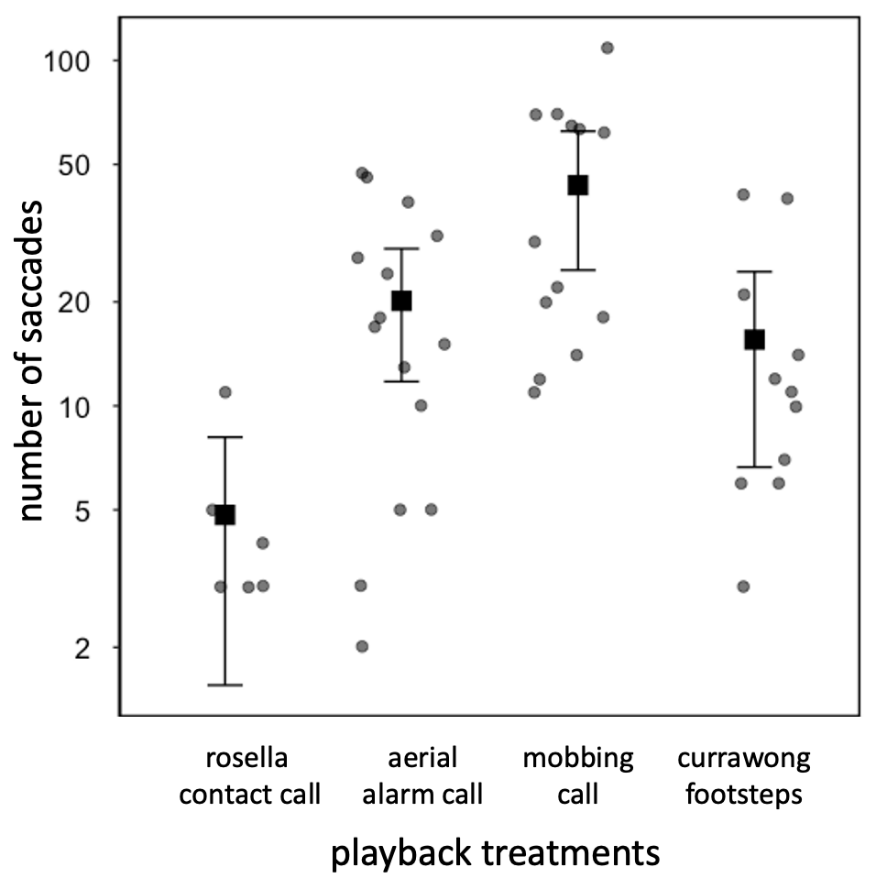
**

**Supplementary Figure 2.** Number of saccades by incubating female scrubwrens responding to different playback treatments, excluding 2 cases where males responded to the mobbing call playbacks. Each round point represents a different focal female; square points and error bars indicate means and 95% CI. Note that the y axis is on a log scale. The results of statistical analysis are shown in Supplementary Table 3; n = 6 for rosella contact calls, n = 15 for scrubwren aerial alarm calls, n = 13 for scrubwren mobbing calls, and n = 11 for currawong footsteps; cases when the focal female either did not respond or fled to the playback are excluded.

**Supplementary Table 3.** Output from LMM investigating the number of saccades by incubating female scrubwrens responding to the playback treatments, excluding 2 cases where males responded to the mobbing call playbacks. Number of saccades is logarithmically transformed. Whether a camera light was turned on and log-transformed response duration were also included as fixed factors. Significant effects are shown in bold. n = 6 for rosella contact calls, n = 15 for scrubwren aerial alarm calls, n = 13 for scrubwren mobbing calls, and n = 11 for currawong footsteps; cases when the focal female either did not respond or fled to the playback are excluded.

| Fixed effects | Estimate ± SE | χ^2^ | df | t ratio | P |
| --- | --- | --- | --- | --- | --- |
| Intercept | 0.834 ± 0.432 |  |  |  |  |
| Camera light (on – off) | 0.044 ± 0.385 |  | 1 | 0.115 | 0.910 |
| Response duration | 0.279 ± 0.149 |  | 1 | 1.871 | 0.069 |
| Treatment |  | 19.596 | 3 |  | **<0.001** |
| Treatment (rosella – aerial) | -0.724 ± 0.544 |  | 1 | -1.332 | 0.550 |
| Treatment (rosella – mobbing) | -1.635 ± 0.532 |  | 1 | -3.072 | **0.021** |
| Treatment (rosella – footsteps) | -0.639 ± 0.562 |  | 1 | -1.136 | 0.671 |
| Treatment (aerial – mobbing) | -0.911 ± 0.282 |  | 1 | -3.232 | **0.017** |
| Treatment (aerial – footsteps) | 0.086 ± 0.301 |  | 1 | 0.285 | 0.992 |
| Treatment (mobbing – footsteps) | 0.996 ± 0.314 |  | 1 | 3.178 | **0.019** |

**Reference**

Magrath, R. D., Leedman, A. W., Gardner, J. L., Giannasca, A., Nathan, A. C., Yezerinac, S. M.,

& Nicholls, J. A. (2000). Life in the slow lane: Reproductive life history of the White-browed Scrubwren, an Australian endemic. Auk, 117(2), 479–489. https://doi.org/10.2307/4089728
